# Supplementary material for: Collaborative intelligence in AI: Evaluating the performance of a council of AIs on the USMLE
Source: PLOS Digit Health. 2025 Oct 9;4(10):e0000787. doi: 10.1371/journal.pdig.0000787 (PMC12510544; doi:10.1371/journal.pdig.0000787)
Supplement: S2 File — (ZIP) [file pdig.0000787.s002.zip › S2 Data Raw data files containing unprocessed question inputs and LLM outputs including transcripts of deliberations/STEP3_Question-52-1_Type-MC-NJ.docx]

**Facilitator** **[Round 1]:** Patient Information Age: 62 years Gender: M, self-identified Ethnicity: white, self-identified Site of Care: office History Reason for Visit/Chief Concern: ""My legs hurt when I walk, and it's getting worse."" History of Present Illness: 3-month history of worsening leg pain, pain exacerbated by walking; peak intensity after 1 block, pain resolves completely with rest, pain rated 4/10 at worst Past Medical History: hypertension, mild angina, type 2 diabetes mellitus Medications: lisinopril, metoprolol, furosemide, glyburide, lovastatin Allergies: no known drug allergies Psychosocial History: has smoked one-half pack of cigarettes daily for 44 years Physical Examination Temp 36.0°C (96.8°F) Pulse 72/min Resp 14/min BP 140/90 mmHg O2Sat - Height 164 cm (5 ft 5 in) Weight 90 kg (198 lb) BMI 33 kg/m² Appearance: no acute distress HEENT: funduscopic shows grade 2/4 arteriovenous nicking Neck: no jugular venous distention Pulmonary: clear to auscultation; mildly diminished lung sounds Cardiac: no bruits; distant heart sounds Abdominal: obese; no tenderness, guarding, masses, bruits, or hepatosplenomegaly Extremities: no joint erythema, edema, or warmth; no hair on toes; no femoral bruits; dorsalis pedis, radial, and femoral pulses intact Neurologic: sensation to vibration intact Question: Which of the following is the most appropriate diagnostic study? (A) Ankle brachial index (B) Arteriography (C) ECG (D) Echocardiography (E) MUGA scan

**AI_A:** RESTATING THE QUESTION: This query asks us to discern the most fitting diagnostic test for a 62-year-old male patient with a clinical picture suggestive of peripheral arterial disease (PAD), induced by walking and relieved by rest. The patient also has risk factors for PAD, including hypertension, diabetes mellitus, and a history of smoking. The potential diagnostic options presented are the ankle brachial index (ABI), arteriography, ECG, echocardiography, and MUGA scan. PLANNING: The key assumptions here are the patient's notable clinical symptoms and his high-risk status due to comorbidities, along with the first-choice diagnostic test for PAD. An alternate assumption would be if the patient's symptoms were rooted in a heart problem, such as stable angina, rather than PAD, given the prominent history of mild angina. CLINICAL REASONING: Looking closely at the options provided, with PAD in our differential diagnosis, ABI (Option A) stands out. The ABI is a simple, non-invasive diagnostic tool that compares the blood pressure in the ankle with the blood pressure in the arm. An ABI of less than 0.9 is diagnostic of PAD. Option B, arteriography, is generally the next step after ABI and is used when an intervention is planned, not for initial diagnosis. Options C, D, and E are all cardiac focused tests and, given the patient's symptomatology focusing on claudication, are less likely to provide the needed diagnosis. CONCLUDING: Patient history, which includes risk factors such as chronic smoking, hypertension, diabetes, absent toe hair (which suggests decreased peripheral blood flow), no femoral bruits, and the symptom of claudication - pain in muscle groups that occurs with a predictable amount of exercise and is relieved by rest - is highly suggestive of PAD. The ankle brachial index is the most appropriate diagnostic test in this scenario. RESPONSE: Therefore, the most appropriate diagnostic study for this patient is (A) the Ankle Brachial Index.

**AI_B:** (1) RESTATING THE QUESTION: The question is seeking the most suitable diagnostic test for a presented case. The patient is a 62-year-old male who experiences pain in his legs when walking which resolves with rest. He has a history of hypertension, mild angina and type 2 diabetes mellitus, and he is a chronic smoker. On examination, there are no hair on his toes, an indicator of poor peripheral circulation. These symptoms lead toward a potential diagnosis of peripheral artery disease (PAD), a condition characterized by narrowing of the peripheral arteries due to atherosclerosis, affecting legs more commonly, uncontrolled hypertension and diabetes, smoking can be the major risk factor. (2) PLANNING: The options presented include ankle brachial index (ABI), arteriography, ECG, echocardiography and MUGA scan. ABI is a simple, non-invasive test used to diagnose PAD by comparing the blood pressure in the ankle with the blood pressure in the arm. Arteriography, a more invasive procedure, involves injection of a contrast medium into the circulation to visualize blood vessels on X-ray. ECG primarily evaluates heart's electrical activity, while echocardiography is used to visualize heart's structure and function. MUGA scan is a type of nuclear imaging test that shows how well the heart is pumping blood. (3) CLINICAL REASONING: Given the symptomatic presentation of the patient, the most probable diagnosis is peripheral artery disease. ABI, a widely accepted and noninvasive diagnostic tool, can confirm the diagnosis by calculating the ratio of blood pressure at ankle and arm. Low ABI indicates PAD. Arteriography being more invasive with risk of allergic reaction or kidney damage from contrast medium, is reserved for cases where ABI is inconclusive or when revascularization is considered. ECG, echocardiography and MUGA scan are primarily for assessing heart conditions, not directly indicated in this scenario. (4) CONCLUDING: Given patient's clinical picture indicative of PAD and the need for a non-invasive diagnostic test that can efficiently detect PAD, ABI appears to be the most appropriate diagnostic tool. Hence, we can assume the best suitable test in this scenario. (5) RESPONSE: The most appropriate diagnostic study for this patient, based on clinical presentation and relevant indications for each of the listed tests, would be (A) Ankle brachial index.

**AI_D:** (1) RESTATING THE QUESTION: The challenge here is to decide the best diagnostic test out of (A) Ankle brachial index (ABI), (B) Arteriography, (C) ECG, (D) Echocardiography, and (E) MUGA scan to confirm the diagnosis for a 62-year-old man presenting with leg pain during movement which suggests a potential vascular problem. (2) PLANNING: The condition sounds suspicious of peripheral arterial disease (PAD). An ABI test, option (A), measures the ratio of the blood pressure at the ankle to the blood pressure in the upper arm which provides an indication of blocked arteries (peripheral vascular disease). Arteriography (B) is a more invasive procedure where a dye is injected into the blood vessels to make them visible under X-rays; it could potentially confirm the diagnosis but is typically reserved when we plan for a vascular intervention. ECG (C) is used to detect heart problems, but this patient's physical examination did not suggest acute cardiac issues, although he has past medical history of hypertension and mild angina. Echocardiography (D) is also for heart issues, and MUGA scan (E) evaluates the function of the heart's ventricles. (3) CLINICAL REASONING: Based on the clinical findings, leg pain on exercise relieved by rest (a phenomenon known as "claudication"), no hair on toes (indicating impaired blood flow to the skin), the smoking history, hypertension, and diabetes mellitus - all these signpost towards PAD. ABI fits well in this patient's context and it is a non-invasive, cost-effective way to diagnose PAD. The other tests, while accurate for cardiovascular related conditions, seem excessive as first-line diagnostic tests for this patient. (4) CONCLUDING: Therefore, initiating a more aggressive and invasive diagnostic test lacks clinical priority. It is more reasonable to start with a less invasive procedure such as the ABI, which relays significant information regarding peripheral arterial function. (5) RESPONSE: The most appropriate diagnostic study for this patient due to his symptoms, personal medical history, and exam findings would be (A) Ankle brachial index.

**AI_C:** (1) RESTATING THE QUESTION: This case presents a 62-year-old white male patient who is experiencing increasing leg pain during walking that resolves with rest. In addition, the patient has a medical history of hypertension, mild angina, and type 2 diabetes mellitus and he smokes half a pack of cigarettes daily. The significant change of his condition is a 3-month history of worsening leg pain, which intensifies after walking 1 block and is rated as 4/10 at its worst. The physical examination showed no hair on his toes and grade 2/4 arteriovenous nicking in fundoscopic examination. The query inquires which diagnostic test would be most suitable to progress with this patient's case? (2) PLANNING: Here, we need to relate the symptoms with the patient's past medical history, habits, and the present physical conditions to identify potential causes of the leg pain. The most likely underlying cause appears to be poor blood flow to the legs which may be attributed to smoking and diabetes, both known to contribute to peripheral arterial disease (PAD). We can investigate this hypothesis through several methods. These mainly include Ankle brachial index (ABI), Arteriography, ECG, Echocardiography, and MUGA scan. Upon evaluation, the ABI, a simple, noninvasive method for diagnosing PAD, seems to be the most clinically relevant and efficient initial approach. (3) CLINICAL REASONING: The patient's symptoms, ie. the leg pain that's worsened by walking and relieved by rest, is indicative of claudication, which is often seen in PAD. PAD is generally caused by atherosclerosis, and given the patient’s history of type 2 diabetes, smoking, hypertension and hyperlipidemia, he is at high risk for atherosclerotic disease. ABI, a test that compares the blood pressure in the ankle to the blood pressure in the arm, is commonly used to screen for PAD as an increase in this ratio may indicate reduced blood flow to the extremities. Other diagnostic options, Arteriography, ECG, Echocardiography, and MUGA scan are not as relevant to PAD as they focus more on the heart and vasculature at large. (4) CONCLUDING: In sum, this 62-year-old patient, with a history of smoking and type 2 diabetes, is presenting symptoms indicating claudication likely due to peripheral arterial disease. The absence of hair on toes signifies poor blood circulation further supporting the suspicion. Therefore, the diagnostic test should primarily aim to substantiate this assumption. (5) RESPONSE: Given the above clinical reasoning, the most appropriate diagnostic study in this scenario is Ankle brachial index - option (A).

**AI_E:** RESTATING THE QUESTION: The clinical inquiry seeks to identify the optimal diagnostic procedure for the symptoms presented by the 62-year-old male patient whose primary complaint is an increasing leg pain that is aggravated by walking and relieved by rest. As diagnostic options, the modalities of 'ankle-brachial index' (a measure of the fall in blood pressure in the arteries going from the upper arm to the lower leg which can indicate peripheral artery disease), 'Arteriography' (an imaging test that uses x-rays and a special dye to see inside the arteries), 'ECG' (an electrocardiogram, which measures the heart’s electrical activity), 'Echocardiography' (a test that uses sound waves to produce images of the heart) and 'MUGA scan' (multiple gated acquisition scan, a type of nuclear imaging test that shows how well the heart walls are moving and how much blood is being ejected with each heartbeat) have been given. PLANNING: Given his history of smoking, hypertension, type 2 diabetes, and mild angina, coupled with the characteristics of his pain (intermittent claudication), the primary assumption is that the patient could be suffering from Peripheral Artery Disease (PAD). This is further supported by the absence of hair on his toes – often a sign of reduced blood flow. One contradiction to this diagnosis is the presence of palpable pulses in his lower extremities. However, the most cost-effective and non-invasive method to verify the PAD assumption would be the 'Ankle-Brachial Index' test. CLINICAL REASONING: While the symptoms presented by the patient could be the effect of multiple conditions, PAD stands out due to his risk profile (smoking, diabetes, hypertension) and the typical presentation of the pain. PAD is a condition where the peripheral arteries (serving the legs, stomach, arms, and head) narrow down due to plaque buildup (atherosclerosis), restricting blood flow. This condition most commonly affects the arteries in the legs. Arteriography, ECG, Echocardiography or MUGA scans would typically be prescribed more for conditions related to the heart, not the peripheral arteries. An ankle-brachial index test directly measures the patient’s blood pressure in the ankle and arm to compare the two, providing an accurate assessment of the blood flow to the affected area. If PAD is indeed the underlying issue, the patient's ankle blood pressure would be lower in comparison to the arm. CONCLUDING: Considering the patient's symptomology and history, the most initially suitable diagnostic study is the Ankle-Brachial Index. It's the recommended first line tool for diagnosing PAD, it's cost-effective, and it's less invasive, making it appropriate for an office setting. However, this doesn't rule out the necessity of other studies such as arteriography, ECG, echocardiography, or a MUGA scan, as the patient's condition and symptoms evolve and become more clear. RESPONSE: Therefore, the most appropriate diagnostic study for this patient, given his symptoms and history, is (A) Ankle Brachial Index.

**Facilitator** The consensus among the AI responses is that the most appropriate diagnostic study for the 62-year-old male patient, who presents with leg pain exacerbated by walking and relieved by rest, is the Ankle Brachial Index (ABI). The patient's symptoms, including the absence of hair on his toes, suggest Peripheral Artery Disease (PAD), a condition often associated with risk factors such as smoking, hypertension, and diabetes mellitus, all of which the patient has. ABI is a simple, non-invasive diagnostic tool that compares the blood pressure in the ankle with the blood pressure in the arm. An ABI of less than 0.9 is diagnostic of PAD. Other diagnostic options such as arteriography, ECG, echocardiography, and MUGA scan are more focused on cardiac conditions and are less likely to provide the needed diagnosis in this scenario. Therefore, the consensus is that the most appropriate diagnostic study for this patient is the Ankle Brachial Index (Option A).
